# Microbial decomposition of keratin in nature—a new hypothesis of industrial relevance

**DOI:** 10.1007/s00253-015-7262-1

**Published:** 2016-01-12

**Authors:** Lene Lange, Yuhong Huang, Peter Kamp Busk

**Affiliations:** Department of Chemical and Biochemical Engineering, Technical University of Denmark, 2800 Lyngby, Denmark

**Keywords:** Fungal and bacterial keratinases, Endo-, exo-, and oligoacting keratinases, Synergistic enzymes, Chemical boosters, Lytic polysaccharide monooxygenases, Keratin decomposition model

## Abstract

Discovery of keratin-degrading enzymes from fungi and bacteria has primarily focused on finding one protease with efficient keratinase activity. Recently, an investigation was conducted of all keratinases secreted from a fungus known to grow on keratinaceous materials, such as feather, horn, and hooves. The study demonstrated that a minimum of three keratinases is needed to break down keratin, an endo-acting, an exo-acting, and an oligopeptide-acting keratinase. Further, several studies have documented that disruption of sulfur bridges of the keratin structure acts synergistically with the keratinases to loosen the molecular structure, thus giving the enzymes access to their substrate, the protein structure. With such complexity, it is relevant to compare microbial keratin decomposition with the microbial decomposition of well-studied polymers such as cellulose and chitin. Interestingly, it was recently shown that the specialized enzymes, lytic polysaccharide monoxygenases (LPMOs), shown to be important for breaking the recalcitrance of cellulose and chitin, are also found in keratin-degrading fungi. A holistic view of the complex molecular self-assembling structure of keratin and knowledge about enzymatic and boosting factors needed for keratin breakdown have been used to formulate a hypothesis for mode of action of the LPMOs in keratin decomposition and for a model for degradation of keratin in nature. Testing such hypotheses and models still needs to be done. Even now, the hypothesis can serve as an inspiration for designing industrial processes for keratin decomposition for conversion of unexploited waste streams, chicken feather, and pig bristles into bioaccessible animal feed.

## Introduction

Keratin is a fibrous and recalcitrant structural protein and is the third most abundant polymer in nature after cellulose and chitin. A wide spectrum of animals (mammals, fish, birds, and reptiles) has developed diversified keratin as a structural part of their outer protection. Keratin is a structural component of skin, hair, feather, horns, hooves, cloves, nails, beaks, reptilian osteoderm, and fish teeth and slime (McKittrick et al. 2012). Keratin renders animals more robust against both abiotic stress and biotic attacks. Since microbial degradation of keratin is not widespread in nature, keratin can serve as an efficient defense even against microbial attack. Keratin is truly recalcitrant. There are many known examples of preserved hair and skin on archeological materials. There are also numerous examples of keratinaceous materials that have passed undecomposed or only partially decomposed through the gut channel system of animals without contributing nutritive value to the animal. However, keratin does not accumulate in nature. It is broken down. The focus of this paper is to create an overview of keratin decomposition mechanisms in nature.

The term keratinase is used to designate the subset of proteases which have keratinolytic activity. The more we study the enzymatic decomposition of keratin, the more obvious it becomes that a distinction between true keratinases and other proteases is not straightforward. Recent findings suggest that several proteases may have keratinolytic activity but that such activity only leads to full keratin decomposition if several different keratinolytic enzymes act together (Huang et al. 2015a; Lange et al. 2014). A conceptual rather than enzymatic comparison to the mechanism of breakdown of cellulose (and chitin) makes good sense. With cellulose, degradation is not the effect of only one enzyme; basically, five enzymes, e.g., GH5, lytic polysaccharide monooxygenases (LPMOs)/AA9, GH6, GH7, and GH3, are needed to break down cellulose (Busk et al. 2014). When considered from this perspective, keratinases may be defined as proteases that have keratinolytic function, which together with other keratinolytic enzymes contribute to keratin decomposition. Further, recent research has provided evidence for that biocatalysis may not act in isolation. Rather, enzymatic and biochemical mechanisms may act synergistically not just for decomposition of lignocellulosic structures (Dashtban et al. 2009) but also for keratin decomposition (Huang et al. 2015a; Lange et al. 2014; Yamamura et al. 2002).

The major part of industrial biotechnology addresses market needs connected with conversion of plant biomass materials. By contrast, upgrade of animal-derived biomass has become a focus of the new bioeconomy to a very limited extent only. However, more attention is gradually being attracted by the value generated by upgrade of for example slaughterhouse waste and by the need to address the growing demand for protein-rich animal feed. Keratinaceous waste streams such as feather and pig bristles provide an interesting and underexploited source of animal feed protein in most parts of the world (Gousterova et al. 2005).

**Fig. 1:**
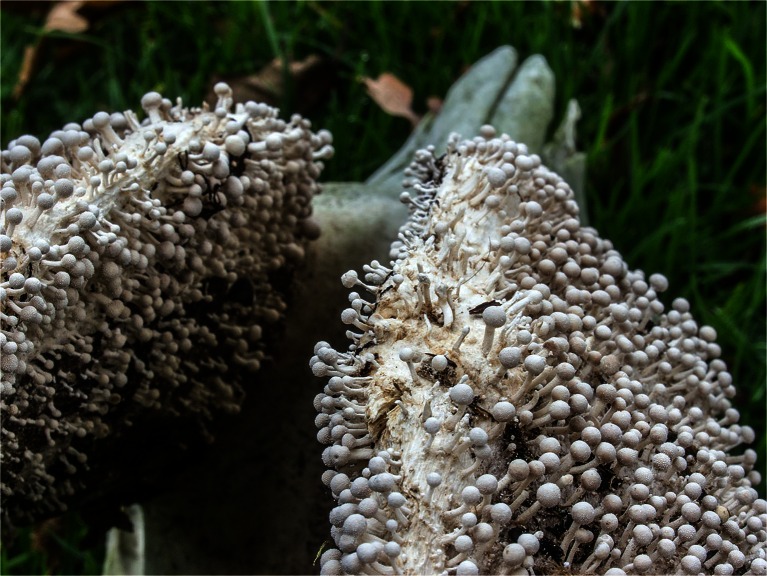
*Onygena corvina* here shown growing on horn; *O. corvina* are also described to grow on other keratinaceous materials in nature, such as e.g. feathers (Lange and Hora 1975)

Most published keratinase reviews focus only on bacterial keratinases (Brandelli 2008; Daroit and Brandelli 2014; Sahni et al. 2015) or include only very limited information about fungal keratinases (Brandelli et al. 2010; Gupta and Ramnani 2006; Gupta et al. 2013; Korniłłowicz-Kowalska and Bohacz 2011; Onifade et al. 1998). However, fungal keratinases play a very important role not just in dermatophytic fungi but also in natural biomass conversion (e.g., *Engyodontium album* (*Tritirachium album*) (Ebeling et al. 1974), *Chrysosporium keratinophilum* (Otcenasek and Dvorak 1964), *Doratomyces microsporus*, *Paecilomyces marquandii* (Gradisar et al. 2000; Gradisar et al. 2005), and *Onygena corvina* (Huang et al. 2015a) (Fig. 1)).

In nature, fungi and bacteria work together in breaking down the recalcitrant and abundant keratinaceous structures and in so doing exploit this rich protein and nitrogen source. The time is therefore ripe for a review which concentrates on the origin, diversity, mechanism, and role of fungal enzymes, where microbial enzymes are examined from the perspective of a synergy between biocatalysis and chemical catalysis in keratin decomposition.

Most published work on industrial processes for degradation of keratin has focused on commercializing and using bacterial keratinases for decomposition of keratinaceous materials (Table 1). However, it appears to be very difficult or maybe impossible to find single-component bacterial enzymes that can do the job alone. No reports have been published on successful bacterial enzyme breakdown of, for example, pig bristles, and even for feather a single bacterial enzyme cannot fully decompose keratin to bioavailable and bioaccessible amino acids and peptides (Laba and Rodziewicz 2014).

**Table 1:** Current commercial keratinolytic proteases

Product name	Source of enzyme	Enzyme function EC number	Merops protein family	Substrates of enzyme product	Example of trade name and provider
Protease P4860	*Bacillus licheniformis*	3.4.21.62	S8	Chicken (leg) bone protein	Alcalase, Novozymes A/S
Protease P5860	*Bacillus* sp.	3.4.21.62	S8	Keratin	Esperase, Novozymes A/S
Protease P3111	*Bacillus* sp.	3.4.21.62	S8	Keratin	Savinase, Novozymes A/S.
Versazyme	*Bacillus licheniformis*	3.4.21.62	S8	Simple and complex vegetable and animal proteins, feather	Versazyme, BioResource International, Inc.
Prionzyme	*Bacillus licheniformis*	3.4.21.62	S8	Prion, keratin	Prionzyme, Genencor International, Inc.
Proteinase k	*Tritirachium album*	3.4.21.64	S8	Prion, keratin	Proteinase K, New England Biolabs

The first step in research to find new fungal enzymes for keratin breakdown, however, is faced with a serious inherent difficulty and a challenge that must be overcome. Screening for keratinases is a very efficient way to unintentionally isolate human pathogens—the fungal dermatophytes—as positive hits in the screening process. The current review will focus on fungal keratinases, produced by non-pathogenic fungi, with the potential for being approved for use in animal feed processing by the regulatory system and also acceptable as regards of workers’ health in the processing industries. The second step in keratinase research is about improving our overall understanding of the mechanism of microbial keratin decomposition in nature. We wish to learn from nature in order to design improved enzymatic keratin decomposition processes; these could involve the use of blends of enzymes and boosting principles, or microbial consortia, composed of non-pathogenic fungi and bacteria, for more efficient keratin breakdown, making this unexploited protein resource available and accessible for animal nutrition.

Much research on keratinases has been done with the objective of understanding the role of keratinases for fungal dermatophytic pathogenesis. This area is also a highly interesting source of insightful information for a conceptual understanding of saprophytic breakdown of keratin (viz., the biotechnology and resource efficiency bioeconomy Waste2Value perspective). There is considerable potential for faster progress, both scientifically and applied, if the biotechnology approach is connected with the dermatophyte research to improve the understanding of the mechanisms of keratin decomposition. Interestingly, molecular work has led to discovery of an application of keratinases in medicine; keratinases can break down misfolded and infectious prion protein, a curative effect not achieved earlier (Langeveld et al. 2003). Such findings may lead to a higher level of keratinase research activity.

Most enzyme studies of keratinases have been built on finding the enzyme by activity testing and characterizing the individual enzymes by protein recovery through purification. In the last one to two decades, the DNA and RNA approach has also been brought in use, first through an EST, expressed sequence tag methodological approach. Only a few articles describe work that has taken full advantage of the options available to rapidly gain increased insight into keratinases through use of explorative and comparative genomics, transcriptomics and mass spectrometry (MS)/proteomics (Huang et al. 2015a; Inada and Watanabe 2013; Lange et al. 2014; Park et al. 2014; Yong et al. 2013). It is to be expected that progress in understanding the molecular structure of a potentially valuable polymer such as keratin will be gained soon, due to increased attention to the need for improved resource efficiency. Such new information will also form a stronger base for increased understanding of what is needed for developing an optimized process for keratin degradation and decomposition.

This review intends to contribute to formulation of new conceptual models for keratin breakdown, which can be of benefit to future efficient and sustainable use of this underexploited protein resource, while also enabling us to develop more efficient approaches for controlling dermatophytes in man and animals. The expected increasingly challenging conditions for agricultural production and the foreseen growth in global population put high demands on improved resource efficiency. This can be achieved by optimizing the use of all parts of agricultural production. Therefore, upgrade of keratinaceous waste to protein-rich animal feed has become a very interesting option to pursue. Furthermore, the more humid and warmer climate in many parts of the world is expected to lead to an increase in dermatophyte infections (Garcia-Solache and Casadevall 2010; La Porta et al. 2008). An increased understanding of the fungal (dermatophyte) mechanisms of keratin degradation is relevant also for control of such serious human and animal diseases. Of relevance for both objectives, upgrade of keratinaceous waste and fighting dermatophytic infections, it is important to learn from the mechanism of microbial breakdown of keratin in nature, degradation by fungi alone, by bacteria alone, or by the combined activity of fungal plus bacterial enzymes.

## Keratin structure

Achievements in understanding the enzymatic decomposition of keratin go hand in hand with understanding the keratin structure, from the microscopic to the biochemical and molecular level.

Keratins are fibrous proteins found in the integument (outer covering) of most vertebrates, reptiles and fish (Meyers et al. 2008). The structural keratinaceous proteins are recalcitrant polymers. The recalcitrance is due to properties such as a high degree of cross-linking by disulfide bonds, hydrogen bonds, and hydrophobic interactions. Based on their secondary structure, keratins are classified into α-keratin and β-keratin. β-Keratin is rich in β-pleated sheets (Meyers et al. 2008) and is constructed from supramolecular fibril bundles (Bodde et al. 2011). α-Keratin consists of α-helical-coil coils which are self-assembled into intermediate filaments (McKittrick et al. 2012; Meyers et al. 2008).

Post-translational modifications of keratin, such as the formation of disulfide bonds, phosphorylation, and glycosylation, can result in diverse types of modified keratin filaments (Yamada et al. 2002). The different keratin characteristics give different degrees of bioaccessibility. Almost all keratinaceous materials (such as feathers, hair, bristles, and wool) possess a mixture of keratins including both α-keratin and β-keratin. Ng et al. (2014) reported that α- and β-keratins are preferentially expressed in different feather parts. It was found that feathers are composed of 41–67 % α-keratins, 33–38 % β-keratin, and also amorphous keratin (Barone et al. 2005; Fraser and Parry 2008). Other keratinaceous materials, such as hair, bristle, and wool, consist mostly of α-keratins (50–60 %), matrix proteins (keratin-associated proteins located in the amorphous space around the intermediate filaments) (20–30 %), and also minor amounts of β-keratins (Daroit and Brandelli 2014). β-Keratin is more accessible for degradation by some keratinases than α-keratin because β-keratin has less disulfide bonds and exhibits the fibril and porosity structure (Gupta and Ramnani 2006). Hairs and feathers belong to what is called hard keratins, based on their function, regulation, and high content of cysteine (Daroit and Brandelli 2014). These hard keratins have diversified morphological structures and numerous disulfide bonds. This makes them insoluble in water, in weak acid and alkaline solutions and in organic solvents, and hard keratins are also resistant to degradation by most protease treatments.

**Fig. 2:**
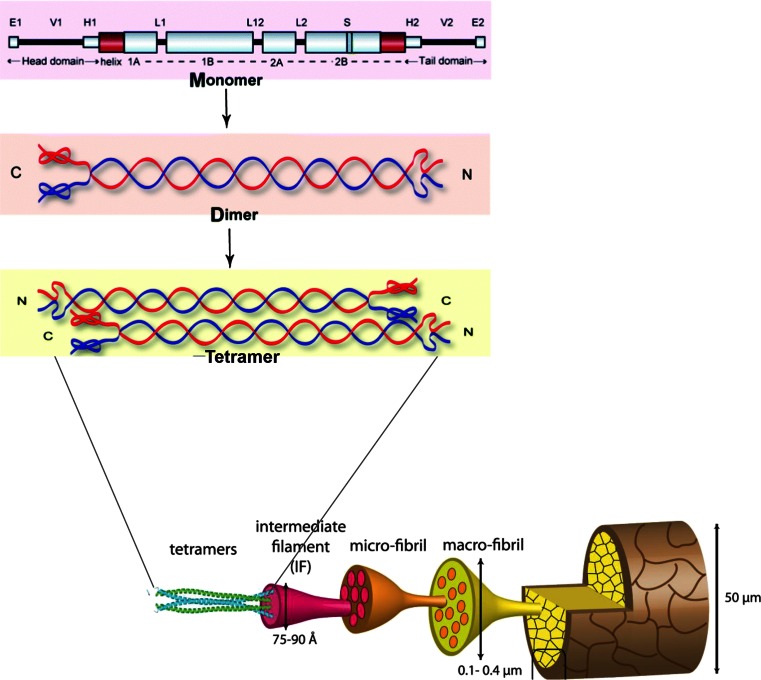
The molecular structure of the detailed composition of hair, modified from Yang et al. (2014) and Banerjee et al. (2014). Permission for re-publishing has been received from copyright owners

The molecular composition of α-keratin is based on two keratin polypeptides, each with a directional head to tail structure, which form a dimeric coiled coil. The α-keratin dimers are formed by self-assembly of such dimeric head to tail polypeptides. Dimers then couple two by two, again by self-assembly, to form the tetramers. Four of these tetramers make up an intermediate filament (McKittrick et al. 2012) (Fig. 2). Structure and self-assembly into intermediate fibers follow a similar pattern in skin and hair (Fuchs 1995). The amino acid composition of the uncoiled head structure includes both threonine and serine (Bragulla and Homberger 2009). Further, the head structure typically has a specific secondary structure due to phosphorylation and glycosylation. The sugar moiety most often found as glycosylation in the head domain of the polypeptide monomer is N-acetyl glucosamine (Berg et al. 2002). Change in the secondary structure in the head region (e.g., due to dephosphorylation and deglycosylation) changes the charge of the keratinaceous protein structure, which may lead to disassembly of the filamentous structure described above (Herrmann et al. 2004; Silengo et al. 2003). Meanwhile, the tail of the keratin also has a crucial role for intermediate filament organization. Sprecher et al. (2001) has confirmed that mutation of the keratin gene, which is related to the variable tail domain, resulted in failure of building of the intermediate filament.

Busk and Lange (2015) reported for the first time the observation that LPMO AA11 genes are found to be consistently occurring in dermatophytic and keratin-degrading fungi. No suggestion was made by Busk and Lange (2015) for how such LPMO AA11 proteins possibly could contribute to breaking down the keratin structure or the keratinaceous matrix components. However, the model presented here of keratin composition and the description of the molecular composition and self-assembly mechanisms (see Fig. 2) may provide basis for suggesting such hypothesis (see section “Suggested mechanism of fungal keratin decomposition in nature” below).

## Methodological approaches in keratinase research

In the process of writing the current Keratinase Review, it is concluded that there is an overall support to the picture of keratin decomposition given by previous review authors (Brandelli 2008; Brandelli et al. 2010; Daroit and Brandelli 2014; Gupta and Ramnani 2006; Gupta et al. 2013; Korniłłowicz-Kowalska and Bohacz 2011; Onifade et al. 1998; Sahni et al. 2015). However, most of the studies cited in such reviews have been done using the living keratinolytic organism and/or the full culture broth to test for the keratin decomposition capabilities of the bacterium or fungus in question (Mazotto et al. 2013). In the next level, we are reviewing here is to study one single monocomponent enzyme at a time. This is most often done by recovering and purifying a single protein from the culture broth (Anitha and Palanivelu 2013; Tork et al. 2013) or by heterologous expression of monocomponent enzyme proteins. However, keratinases have been recombinantly expressed only rather recently, most often in *Escherichia coli* and in the yeast *Pichia pastoris* (Gunkel and Gassen 1989; Hu et al. 2013; Huang et al. 2015a; Lange et al. 2014).

Most research papers and commercialization efforts have aimed at identifying one organism or one main enzyme which can decompose keratin (Table 1). This approach—one gene, one protein, one product—has been the predominant business model for industrial biotechnology for decades. However, this began to change with start of the biomass conversion biorefinery business. In this sector, more than one enzyme was needed for breaking down the lignocellulosic materials to make monomer sugars available for yeast fermentation for bioethanol production or for producing other microbial products such as new materials or biochemical building blocks. The new and more efficient solution for producing several monocomponent fungal enzymes, for conversion of complex and recalcitrant lignocellulose, was to develop one production strain carrying several enzyme genes and producing several enzymes in one and the same fermentation. This made it possible to recover an enzyme blend which could be used for biomass conversion, ready for commercial sale and biorefinery use. Parallel to this, research efforts within keratin decomposition have been made to find out which enzymes are needed and which co-factors or boosting principles may add to the enzymatic break down of keratin. Sulfite has been especially highlighted for this purpose (Grumbt et al. 2013). Rather recently, several papers have been published which report keratinase activity that disappears when recovered from the intact (multi enzyme) culture broth. This finding confirms the interpretation that one purified enzyme alone is not able to decompose fully the recalcitrant keratin structure (Inada and Watanabe 2013; Ramnani and Gupta 2007).

Assaying for keratinase activity has been done by observing breakdown of real item materials (feathers, hair, skin, bristles) (Balaji et al. 2008; Huang et al. 2015a; Lange et al. 2014; Prakash et al. 2010). In vitro assays have been done primarily with azure-tagged sheep’s wool (Keratin azure) which mainly includes α-keratin (Scott and Untereiner 2004) as substrate. Riffel et al. (2003) also synthesized a keratinase substrate consisting of azokeratin coupled with a diazotized arylamine and feather (rich in β-keratin). New efforts are needed in developing keratinase assays that detect both α- and β-keratinase activity. Furthermore, there is a high demand for standardized models for experimental work that uses several keratinase active enzymes together and also for testing such enzyme blends in combination with various boosting principles.

Most keratinase research has been conducted on wild-type bacteria and fungi. To solve the puzzle of what mixture of biocatalysts (enzymes) and abiotic components (e.g., sulfite) are needed to break down different types of keratin, we need to have purified enzyme proteins and monocomponent proteins, recombinantly expressed, available in order to do the right experiments. Further, well-characterized, real-item, representative keratin substrates to work with would also be a great advantage; this would be especially useful if the substrates were representative for the two biggest waste components, feather and bristles.

Recently, omics technologies have been combined to understand more comprehensively how keratin can be degraded by a blend of keratinolytic proteins. *Bacillus* sp. and *Chryseobacterium* sp. are among the best characterized candidates for efficient keratin degradation. The genome sequences of *B. subtilis* strain S1-4 and *Chryseobacterium* sp. strain P1-3 suggested multiple extracellular proteases and keratinases (Park et al. 2014; Yong et al. 2013). The thermophilic bacterium, *Meiothermus ruber*, also has a remarkable ability to degrade chicken feathers. The sequencing of the genome of this strain paved the way for finding new enzyme candidates for degradation of feathers (Inada and Watanabe 2013). However, based on the genome sequence information, it is very hard to identify putative keratinases because the genome usually includes around 100–200 or even more proteases belonging to different protease families (http://merops.sanger.ac.uk/index.shtml). The secretome composition of a keratinolytic microbial strain gives information about which proteins are induced and expressed when the strain is grown on the keratinaceous materials. Activity testing of the culture broth similarly gives information about the protein functions represented in the secretome.

Many species of human pathogenic fungi mainly secrete endoproteases such as aspartic proteases of the Merops A1 and A4 family, serine proteases of the subtilisin family (S8), chymotrypsin-like protease in S19 family, and metalloproteases of two different families (M35 and M36). Such dermatophytic fungi also secrete exoproteases such as aminopeptidases (M28), carboxypeptidases (S10), and dipeptidyl-peptidases (S9) (Monod et al. 2002). Monod et al. (2005) found that two leucine aminopeptidases of the M28 family and two dipeptidyl-peptidases of the S10 family were produced by *Trichophyton rubrum* in a medium with keratin as sole carbon and nitrogen source. Recently, Lange et al. (2014) and Huang et al. (2015a) have employed integrated use of genomics, bioinformatics, MS identification of proteins and activity testing of fractions of culture broth to reveal which minimum blend of proteases of the non-pathogenic ascomycete *O. corvina* is capable of decomposing keratin (see section “Suggested mechanism of fungal keratin decomposition in nature” below).

## Overview of characterized and commercialized bacterial keratinases

Molyneux (1959) was the first to isolate bacteria that are able to degrade keratin. Lin et al. (1992) then were the first to purify and characterize keratinase of the S8 protease family (MEROPS database: http://merops.sanger.ac.uk/index.shtml) from *Bacillus licheniformis* strain. Several more keratinases found at an early stage of the keratinase research were shown to be subtilisin-like proteases, belonging to the serine proteases (S8 family), mainly from *Bacillus* sp. and *Streptomyces* sp. (Brandelli et al. 2010). So the keratinases were defined as serine proteases from the beginning.

Recently, a broader diversity of keratinolytic bacteria has been found with high potential for degrading keratinaceous waste materials. This resulted in identification of different types of keratinases. A keratinase Q1 enzyme from *Chryseobacterium* sp. kr6 was purified and characterized as a member of the M14 metalloprotease family (Riffel et al. 2007). Metalloproteases were also found to be able to degrade the keratin from *Streptomyces* sp. 594 (De Azeredo et al. 2006), *Lysobacter* NCIMB 9497 (Allpress et al. 2002; Wang et al. 2008), *Chryseobacterium* sp. (Silveira et al. 2012; Wang et al. 2008), *B. subtilis* MTCC (9102) (Balaji et al. 2008), *Microbacterium* sp. strain kr10 (Thys and Brandelli 2006), and *Pseudomonas aeruginosa* (Lin et al. 2009). These metalloproteases were shown to be sensitive to EDTA inhibition. However, such keratinolytic metalloproteases are still not included with full information about family and function in the MEROPS database.

Currently, seven bacterial keratinolytic proteases have been commercialized by several different companies (Table 1). All bacterial enzymes in Table 1 are from *Bacillus* species and classified as serine endopeptidases of the subtilisin-type belonging to the S8 protease family with a preference to cleave after hydrophobic residues. Interestingly, as these enzymes efficiently degrade other proteinaceous substrates than keratin they have great commercial value in the detergent industry, in food processing, e.g., degradation of slaughterhouse waste and in the leather industry and other industrial applications (Brandelli et al. 2010; Gupta et al. 2013). However, the currently commercially available keratinases are limited to applications that require a protease working at neutral to high alkaline pH.

Although keratinases are potent and important industrial enzymes, the development of commercially viable decomposition of keratinaceous material such as feather, hair and hoofs has been slow and difficult. This must be ascribed to the complex structure of keratinaceous material, holding a diversity of insoluble networks of different cross-linked proteins. Hence, efficient degradation of keratin in an industrial process may require a blend of different keratinases and possibly other enzymes that attack post-translational modifications of the keratin such as disulfide bridges and glycosylation.

## Overview of fungal keratinolytic enzymes

The most recent work on fungal keratinases is the investigation of the keratinases of the ascomycete *O. corvina* (Huang et al. 2015a; Huang et al. 2015b; Lange et al. 2014). The interesting effect of *Onygena* keratinases on both feather and hair/pig bristles was documented for *O. corvina*. Three *Onygena* species, *O. corvina*, *O. piligena*, and *O. equina* (belonging to the fungal ascomycetous order Onygenales), were found to grow specifically on keratinaceous substrates (Table 2). Based on this, it was concluded that *O. piligena* and *O. equina* could also have high keratinolytic activity. Keratinases from pathogens such as fungal dermatophytes cannot be used for applied purposes due to safety and regulatory issues. But the fungal order Onygenales includes several dermatophytic species. It was therefore important to document that the keratinolytic proteases of *O. corvina* were different from the proteases of the dermatophytic Onygenales. Busk and Lange (2015) have provided strong evidence for that by using the Peptide Pattern Recognition (PPR) to comparatively analyze sequences of a spectrum of Onygenales species, it was possible to identify patterns of conserved peptides, which enable proteases/keratinases from dermatophytes to be distinguished and differentiated from proteases/keratinases from non-pathogenic species. Such analysis was made possible only by the use of the non-alignment-based PPR technology platform, which groups and characterizes enzymes according to predicted function based on their pattern of conserved peptides (Busk and Lange 2015).

**Table 2:** Taxonomic overview of keratinolytic fungi

Order	Genus	Reported species	Ecological niche/substrate affinity	References
	*Onygena*	*O. corvina*, *O. piligena*, *O. equina*	Hair, feather, bristle, horn and hoof	(Currah 1985; Huang et al. 2015a; Lange and Hora 1975)
	*Chrysosporium*	*C. keratinophilum*, *C. indicum*	Hair, sewage sludge	(Currah 1985; Dozie et al. 1994; Rajak et al. 1991; Ulfig et al. 1996)
	*Arthroderma*	*A. gypseum*, *A. otae*, *A. benhamiae*	Hair, sewage sludge, hoof and horn	(Burmester et al. 2011; Busk and Lange 2015; Currah 1985; Staib et al. 2010; Ulfig et al. 1996)
	*Microsporum*	*M. canis*, *M. gypseum*	Pig, sewage sludge, hair, stratum corneum and nail	(Currah 1985; Descamps et al. 2003; Jousson et al. 2004; Ulfig et al. 1996; Vermout et al. 2007)
	*Coccidioides*	*C. immitis*, *C. posadasii*,		(Busk and Lange 2015)
	*Gymnoascoideus*	*G. petalosporus*	Hair	(Rajak et al. 1991)
	*Trichophyton*	*T. rubrum*, *T. tonsurans*, *T. verrucosum*, *T. mentagrophytes*, *T. schoenleinii*, *T. vanbreuseghemii*, *T. terrestre*, *T. ajelloi*	Hair, sewage sludge, skin stratum corneum and nail	(Burmester et al. 2011; Busk and Lange 2015; Chen et al. 2010; Currah 1985; Jousson et al. 2004; Tarabees et al. 2013; Ulfig et al. 1996; Zaugg et al. 2008; Zaugg et al. 2009; Zhang et al. 2014)
Eurotiales	*Aspergillus*	*A. fumigatus*, *A. oryzae*, *A. parasiticus*, *A. niger*, *A. flavus*, *A. terrus*, *A. sulphureus*	Feather, poultry soil, nail and soil	(Anitha and Palanivelu 2013; Farag and Hassan 2004; Friedrich et al. 1999; Kim 2003; Mazotto et al. 2013; Santos et al. 1996; Sousa et al. 2015)
	*Talaromyces*	*T. trachyspermus*	Hair	(Rajak et al. 1991)
	*Paecilomyces*	*P. marquandii*	Stratum corneum and nail	(Gradisar et al. 2005)
Microascales	*Doratomyces*	*D. microsporus*	Stratum corneum and nail	(Gradisar et al. 2000; Gradisar et al. 2005)
	*Scopulariopsis*	*S. brevicaulis*	Hair and poultry farm	(Anbu et al. 2005; Rajak et al. 1991)
Hypocreales	*Myrothecium*	*M. verrucaria*	Feather	(Moreira-Gasparin et al. 2009)
	*Tritirachium*	*T. album*	Horn chips	(Ebeling et al. 1974)
	*Trichoderma*	*T. atrvoviride*		(Cao et al. 2008)
Saccharomycetales	*Candida*	*C. albicans*, *C. tropicalis*	Feather	(Busk and Lange 2015; Lin et al. 1993)
	*Geotrichum*	*G. candidum*	Hair	(Rajak et al. 1991)

The most comprehensive overview of the ecology and substrate associations of the fungal order Onygenales is given by Currah (1985) (see also in Table 2). However, beyond phenotypic descriptions, only very few of these numerous and diversified fungal species have been studied further in any detail with regard to morphology, species characteristics, and habitat associations. It is noteworthy that many of the species described in the Onygenales monograph by Currah (1985) actually grow specifically on keratinaceous substrates in nature. From Currah (1985), we can therefore deduce that a much greater diversity of fungal keratinases is to be found within this group of fungi.

Rajak et al. (1991) described the ability of five different fungal species which are able to digest human hair. The ascomycetous fungus *Gymnoascoideus petalosporus* was found to be the species with the maximum keratin decomposition effect (Table 2).

Dozie et al. (1994) found culture broth of the ascomycete *C. keratinophilum* to have keratin degradation effects even under alkaline and high temperature conditions. Such effects could be demonstrated from the broth itself without the fungus being present. Similarly, culture broth of *Aspergillus fumigatus* was shown to be able to degrade chicken feather even after heating up to 70 °C, and the optimum temperature for keratin degradation was 45 °C (Santos et al. 1996).

Fungal sulphitolysis has been described for the dermatophyte *Microsporium gypseum* by Kuhnert (1992). Sulphitolysis of proteins is one of the basic characteristics of fungal dermatophyte degradation of keratin. The disulfide bonds are cleaved first, and the keratin denatured, giving easy access for proteases/keratinases to degrade the keratin protein even further.

Other research efforts focused on the keratinolytic capability of other types of fungi belonging to, for example, the Eurotiales (*Aspergillus* sp., *Talaromyces* sp., and *Paecilomyces* sp.), Microascales (*Daratomyces* sp. and *Scopulariopsis* sp.), Hypocreales (*Myrothecium* sp., *Tritiachium* sp. and *Trichoderma* sp.), and Saccharomycetales (*Candida* sp. and *Geotrichum* sp.) (see references in Table 2). A comparative overview of similarities and differences of fungal keratinases from Eurotiales, Hypocreales, Onygenales, Microascales, and Saccharomycetales has not yet been compiled. Production of keratinases by other parts of the fungal Kingdom (Basidiomycota, Zygomycota, and Chytridiomycota) has not yet been elucidated.

Keratinases from non-pathogenic fungi have great potential for animal feed applications. However, until now, only a few of such fungal keratinolytic proteases have been characterized (Table 3). Most of the characterized fungal keratinases are from the S8 family of serine protease, such as the proteinase K-like proteases from *D. microsporus* and *P. marquandii* (Gradisar et al. 2005) and endoprotease from *O. corvina* (Huang et al. 2015a; Lange et al. 2014). Lange et al. (2014) and Huang et al. (2015a) first reported that metalloproteases such as M28 and M3 also play an important role in keratin degradation.

**Table 3:** Characterized keratinolytic proteases from non-pathogenic fungi

Microorganism	Enzyme	Merops family	Subgroup (PPR)	Reference
*O. corvina*	Endoprotease 6877	S8	16	(Huang et al. 2015a; Lange et al. 2014)
*O. corvina*	Endoprotease 11652	S8	39	(Huang et al. 2015a; Lange et al. 2014)
*O. corvina*	Exoproteases 8025	M28	3	(Huang et al. 2015a; Lange et al. 2014)
*O. corvina*	Exoproteases 6432	M28	64	(Huang et al. 2015a; Lange et al. 2014)
*O. corvina*	Oligopeptidases 8393	M3	17	(Huang et al. 2015a; Lange et al. 2014)
*T. album*	Proteinase K	S8	–	(Ebeling et al. 1974)
*D. microsporus*	Keratinase (proteinase K)	S8	–	(Gradisar et al. 2005)
*P. marquandii*	Keratinase (proteinase K)	S8	–	(Gradisar et al. 2005)
*C. tropicalis*	Aspartic protease	Aspartic protease (family unknown)	–	(Lin et al. 1993)
*A. parasiticus*	Keratinase	S8	–	(Anitha and Palanivelu 2013)
*A. oryzae*	Keratinase	S8	–	(Farag and Hassan 2004)
*S. brevicaulis*	Keratinase	Serine protease (family unknown)	–	(Anbu et al. 2005)
*M. verrucaria*	Keratinase	Serine protease (family unknown)	–	(Moreira-Gasparin et al. 2009)
*T. atroviride*	Keratinase	Serine protease (family unknown)	–	(Cao et al. 2008)

## Suggested mechanism of fungal keratin decomposition in nature

The conceptual understanding that keratin breakdown may require more than just one enzyme arose with the observation that the keratinase activity of a culture broth disappeared after purifying a keratinase active protein (Inada and Watanabe 2013; Ramnani and Gupta 2007). The concept was iteratively developed further from experiments which indicated that two enzymes work synergistically together in decomposition. The next step was achieved when experimental work provided evidence that disulfide reductase and the intracellular enzyme cysteine dioxygenase can break down sulfur bridges. Cysteine dioxygenase also leads to production and secretion of sulfite; such sulfite is documented to add to decomposition of keratin by breaking the sulfur bridges, and thus giving the enzymes improved access to the keratinaceous substrate (Yamamura et al. 2002).

Study of the non-pathogenic fungus *O. corvina*, colonizing feather, hooves, and horn in nature, added further to the understanding of the fungal mechanism for breaking down keratin. Through this work, evidence was provided for the need for a combination of endoprotease, exoproteases, and oligopeptidase (in S8, M28, and M3family, respectively) to bring about keratin degradation. Recently, a further step forward has been achieved through PPR analysis of a broad spectrum of fungal genomes. The analysis, surprisingly, revealed that the newly discovered grouping of auxiliary proteins, the LPMOs AA11, was found not just in bacteria and in fungi, breaking down chitin, and cellulose and hemicellulose, respectively, but also in the genome of dermatophytic fungi. Furthermore, we have found that LPMO-encoding genes (AA11 subfamily 1) are also present in the genome of *O. corvina*. The other closely related *Onygena* species (e.g., *O. equina* and *O. piligena*) have been reported also to grow on an entire spectrum of keratinaceous materials, containing both α- and β-keratin.

**Fig. 3:**
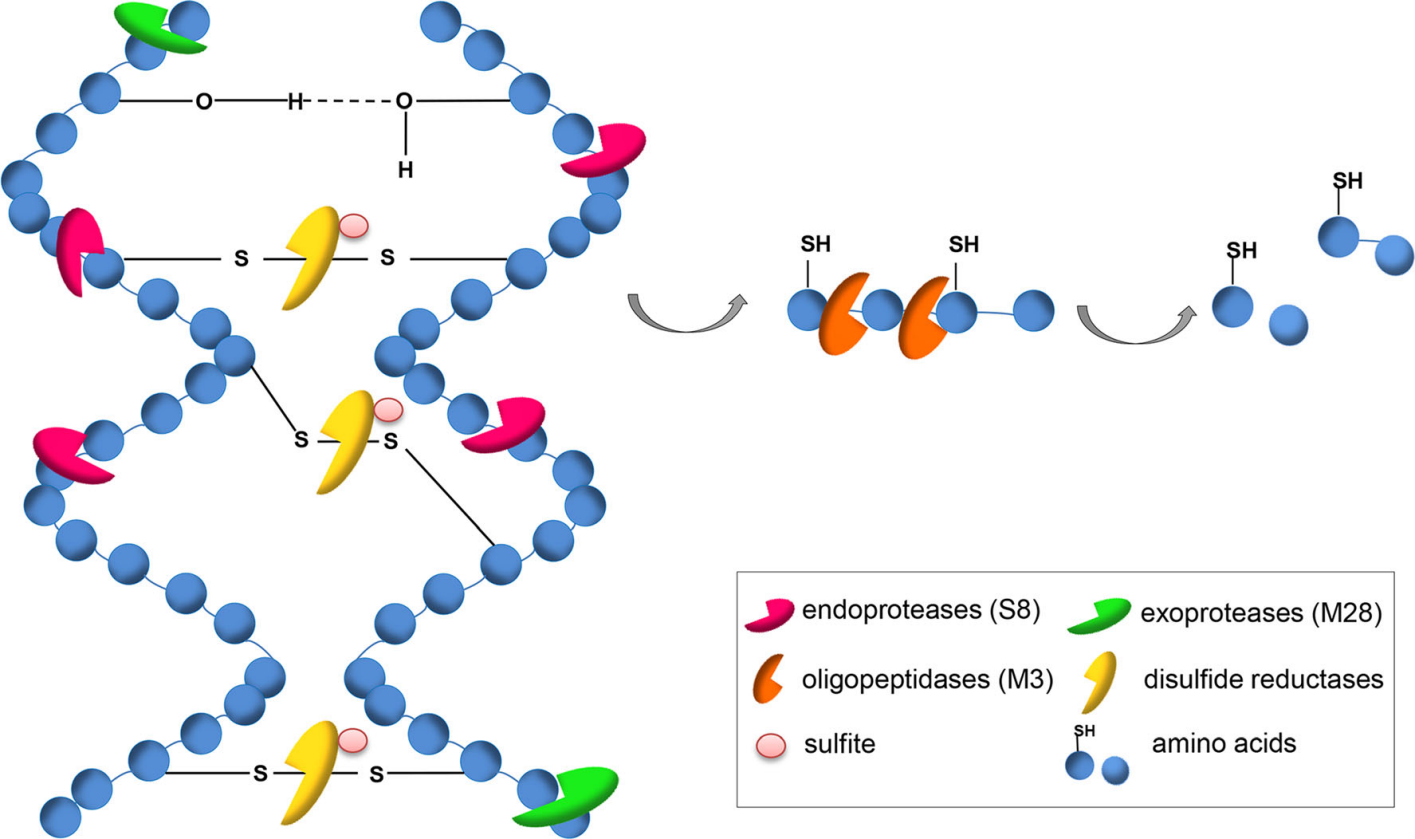
The proteinaceous structure of keratin can be decomposed by a synergistic effect of three proteases: excellular endoproteases (S8), exoproteases (M28), oligopeptidases/metalloproteases (M3), and sulfite/disulfide reductases

Lange et al. (2014) and Huang et al. (2015a) identified the non-pathogenic asomycetous fungal species *O. corvina* (Onygenales) as the preferred model for studying fungal keratinases, based on its physiology, substrate affinity and preferred habitat in nature, where it grows exclusively on keratinaceous materials. These studies included integrated use of genomics, secretomics, and activity profiling of individual fractions of fractionated culture broth of *O. corvina.* The results of such integrated studies were combined with MS analysis of the active fractions of the *O. corvina* culture broth which enabled us to identify which keratinolytic proteases from *O. corvina* were needed as the minimal composition for breaking down the keratinaceous materials. The culture broth was fractionated and the enzyme composition of the most keratinolytically active fractions was identified by matching the MS spectrum of such fractions with the sequences of the proteases found in the *O. corvina* genome (Huang et al. 2015a; Lange et al. 2014). The five proteases identified were shown to belong to three protease families, S8, M28, and M3. The conclusion here was that the keratinolytic effect on pig bristles can be achieved by applying a blend of three specific proteases, S8, M3, and M28. Lastly, it was experimentally documented that the enzyme blend with these three proteases, one from each family, was sufficient for breaking down bristle keratin. Confirmation of this result using a blend of three monocomponent *O. corvina* keratinases, belonging to protease family S8, M3, and M28, is still pending. The conclusion is that the results from the *O. corvina* investigation suggest that a blend of fungal keratinases—an endoprotease (S8), exoprotease (M28), and an oligopeptidase/metalloprotease (M3)—may act synergistically to break down pig bristle keratin (see Fig. 3). For bacterial keratinases, the M28 may be substituted by a bacterial exopeptidase with a similar function to that of the M28 proteases in fungi.

**Fig. 4:**
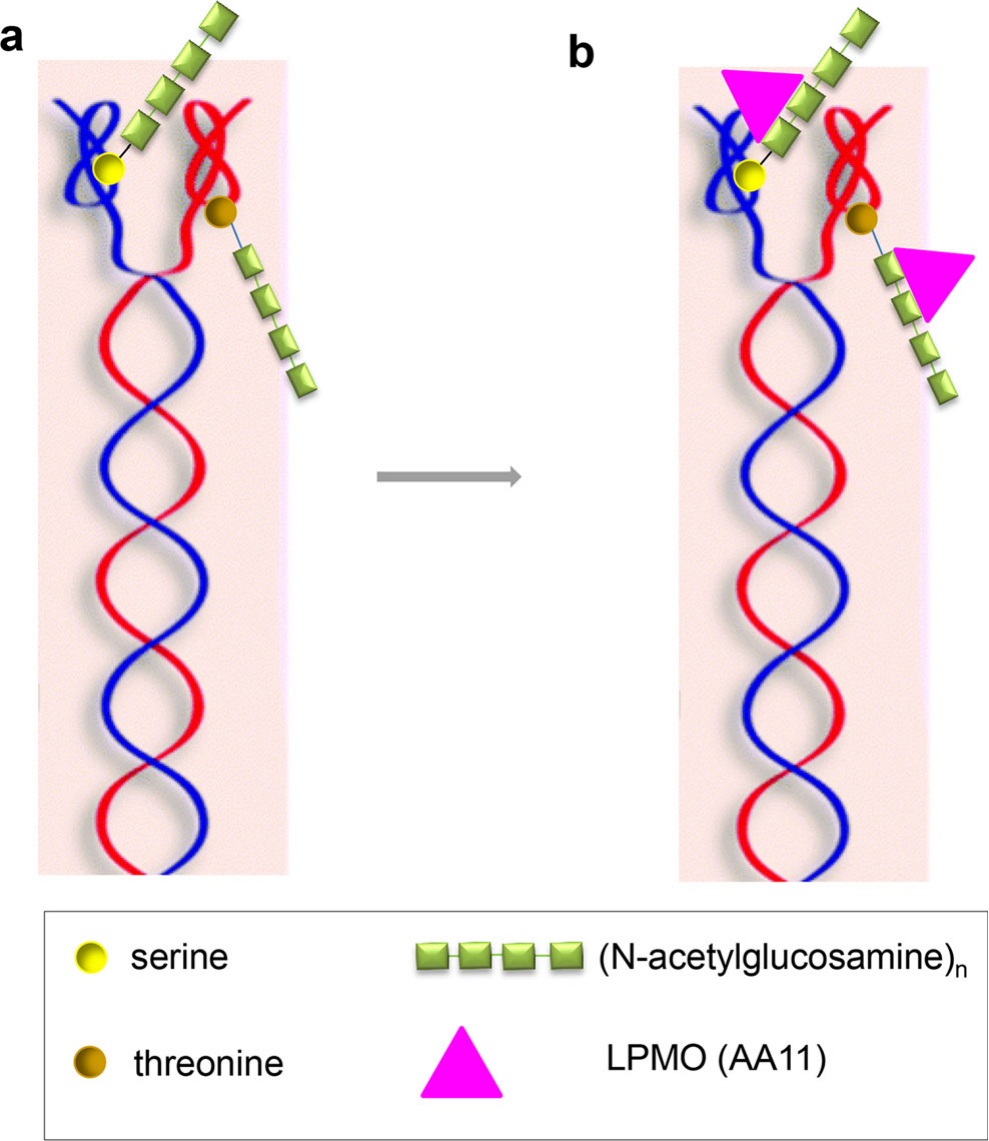
Hypothesis: LPMOs (AA11) break the β-1,4-bonds between N-acetylglucosamine moieties in the glycosylation of serine and threonine in the non-coiled head structure of the keratin filaments; this leads to changes in the charge of the keratin filament head structure (and possibly also of the tail structure (Sprecher et al. 2001)); eventually this causes the de-assembly of the intermediate (self-assembled) keratin filaments. Permission for re-publishing has been received from copyright owners

**Fig. 5:**
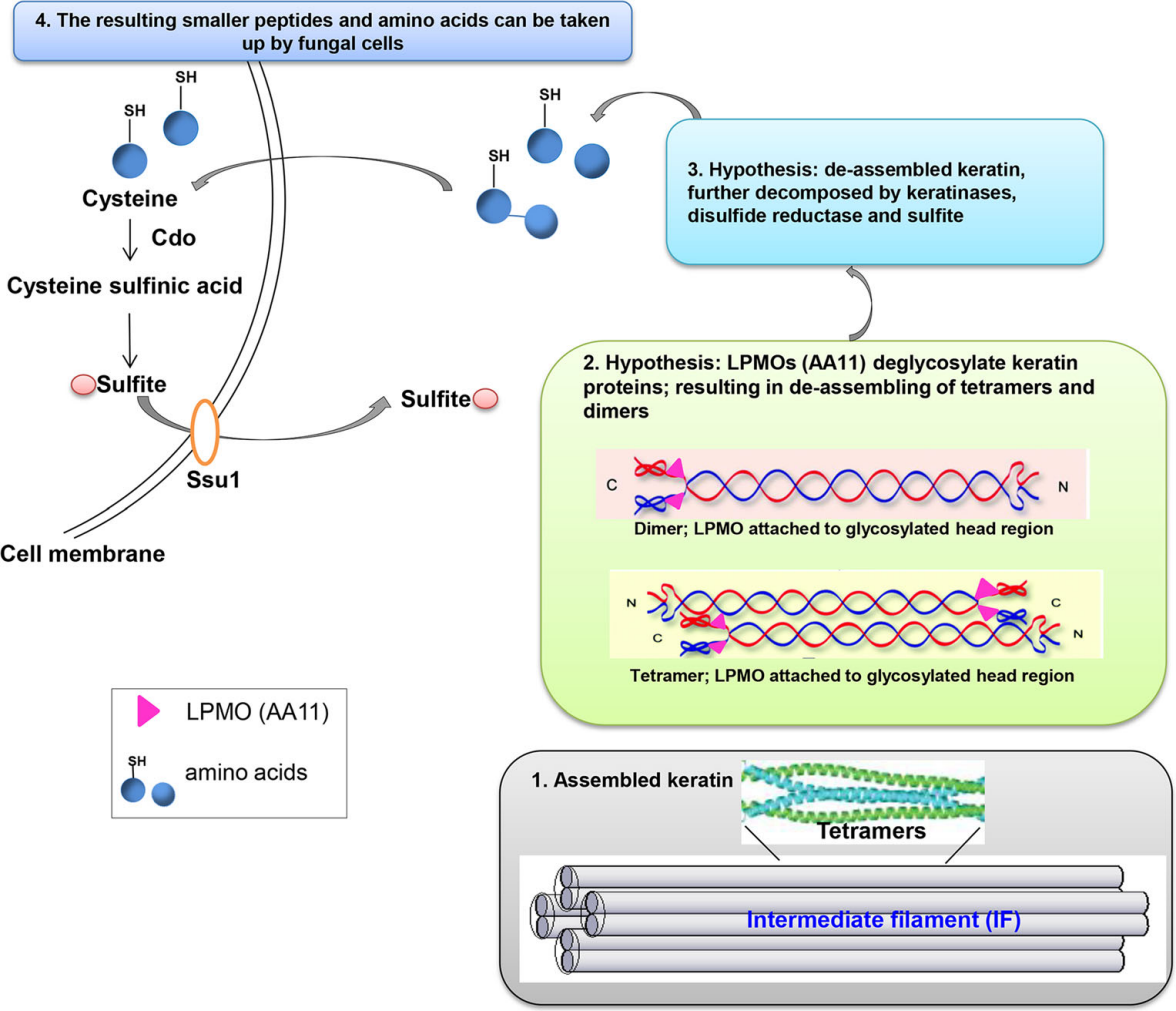
Overview (*1*–*4*) of proposed hypothesis for microbial degradation of α-keratin: *1* gives the structure of assembled α-keratin. *2* LPMOs (AA11) break the glycosylation bond leading to change of steric formation and charge, which again lead to de-assembly of the keratin filaments. *3* The de-assembled keratins are degraded by the activity of three synergistic proteases and sulfite/disulfide reductases as described in Fig. 3. *4* The resulting smaller peptides and amino acids can be taken up by the bacterial or fungal cells. Permission for re-publishing has been received from copyright owners

Besides a blend of three proteases, four additional components have been identified to act in synergy with the keratinase blend treatment and speeding up the degradation process: there are *O. corvina* AA11/LPMOs, disulfide reductase, cysteine dioxygenase, and sulfite (see Fig. 4 and 5).

Busk and Lange (2015) observed for the first time that LPMOs were present in the genomes of a wide selection of dermatophytic fungi and likewise also present in the genomes of non-pathogenic keratin-degrading fungi. This was a very surprising observation because the LPMO proteins, AA9, AA10, and AA11, had so far exclusively been found associated with decomposition of polysaccharides such as cellulose, chitin, hemicellulose and starch.

Based on recent literature describing the latest evidence-based models of keratin molecular composition and structure (Banerjee et al. 2014; Yang et al. 2014), the following two hypotheses for the possible role of AA11 LPMO in keratin-degrading fungi have been formulated.

Hypotheses for LPMO mode of action, contributing to keratin decomposition:

The AA11 LPMOs in keratin-degrading fungi break the glycosylation bonds between N-acetylglucosamine and serine and threonine in the non-coiled head structure of the keratin filaments. This leads to a change of the steric conformation and/or of the charge of the head/monomer. Such changes lead to a loosening of the keratin structure or even to de-assembly of the keratin filaments, as essential parts of the structure of the dimer, tetramers which have been shown to have the capacity of self-assembly have been changed (Bragulla and Homberger 2009; Grumbt et al. 2013) (see Fig. 4, LPMO activity, Fig. 5 (hypothesis overview), and Fig. 2, keratin structure). An alternative hypothesis could be that the LPMOs act directly on the keratin protein, e.g., with tyrosine as substrate. A reaction of monoxygenases on tyrosine is well known (Toyo-oka et al. 2003). This hypothesis cannot be ruled out completely. However, the monoxygenases known for, e.g., tyrosine modification does not lead to a peptide bond breakage, and may not lead by itself to a significant step towards keratin decomposition. Further, it is a type of reaction which is outside the substrate so far found to embrace the activities of the LPMOs.

Recent descriptions of LPMO activity on cellulose and other carbohydrate substrates (Vermaas et al. 2015) suggest that substrate specificity is not as strict as first interpreted. The use of an electron donor to generate active oxygen may act efficiently on many different types of substrate; the mode of action could be that different oxidized species introduce steric effects that disrupt local crystallinity and in some cases allow for polymer decrystallization (Vermaas et al. 2015)).

## Future research and development

More research efforts are needed within the field of characterization of keratinases and to resolve the mechanisms of keratin degradation in nature. Keratinases originating from non-pathogenic fungal species in particular are to a large extent unexplored and unexploited. An obvious initial focus is to characterize and compare the keratinases of fungi belonging to the Eurotiales, Onygenales, and Hypocreales (see Table 2). Maybe optimization of keratinase blends of fungal proteases could be achieved by combining keratinases from these three fungal orders, taking the best enzyme producer from each order. Second, research efforts are needed that use combinations of keratinases of both fungal and bacterial origin. In this way, it may be possible to discover consortia composed of both types of organisms, which are responsible for synergistic keratin decomposition in nature. The third area of investigation, study of the composition of efficient microbial consortia and clarification of the interactions between the consortia members, can be guided by the designs found for keratin decomposition in nature; this is a highly interesting field, both with regard to understanding biomass conversion in nature and for improving industrial upgrade of keratin waste and thus resource efficiency.

In order to benefit the most from the concerted efforts of the research community and gain not just data but also new information and increased conceptual understanding, a set of experimental tools standardized to a higher degree than is the case today would be very valuable. For example, standardized feather and bristle pretreatment regimes are needed prior to enzymatic treatment. There is also a requirement for standardized assays (including assays differentiate eg between decomposition of α- and β-keratin), standardized substrates, and a standardized approach to characterizing function and effect of enzymatic blends and booster principles.

The entire field of the nutritional value for animals of protein-rich feed supplement made from keratinaceous waste needs to be revisited and fully characterized and understood. Much of the data available regarding amino acid profiles, bioaccessibility, and nutritional value is impacted negatively by the pretreatment used (destroying the more complex amino acids). As a result, the keratin animal feed meal used de facto is a blend of smaller granules of intact recalcitrant keratin materials and accessible proteinaceous materials, where the amino acid profile has been lowered in nutritional value through processing. Only a small part of the keratin material is usually fully decomposed in the processes available today. Standardization and accessible, well-characterized samples to be used as benchmarking for new enzyme and enzyme blend discoveries would be highly beneficial. But most importantly, the nutritional value and potential of the keratin-derived animal feed must be fully elucidated.

There are two main drivers for the research suggested above: (1) Interest also in understanding the mechanisms of biomass composition of animal origin. Lignocellulose has been intensely studied, but much less attention has been paid to the animal biomass. (2) The need and demand for additional animal feed proteins, preferably making good use of already existing resources which currently only go to waste. Greater exploitation would achieve increased and improved resource efficiency, to the benefit of the environment, as well as feeding a growing population and creating new jobs in the rapidly growing waste handling industries sector.

Use of keratinases in modern medicine may prove to have much broader potentials: First, the recent discovery of the potential of keratinases in breaking down and inactivating misfolded prion proteins (Langeveld et al. 2003) could prove to be very important for future treatments, especially if the keratinase used in prion research is contributing to understanding prion pathogenicity and to possibly connecting prions with certain types of dementia (Narayan and Dutta 2005). Secondly, LPMOs have been suggested to be a part of the pathogenesis of important human pathogens, e.g., cholera (Loose et al. 2014; Paspaliari et al. 2015).
